# Immune Checkpoint Inhibitor-Related Aortitis Treated with Nivolumab for Esophageal Squamous Cell Carcinoma

**DOI:** 10.70352/scrj.cr.25-0177

**Published:** 2025-06-27

**Authors:** Norihiro Akimoto, Tsutomu Sato, Sho Sato, Hayato Watanabe, Akikazu Yago, Kohei Kasahara, Kenki Segami, Yusuke Suwa, Masakatsu Numata, Takafumi Kumamoto, Aya Saito

**Affiliations:** 1 Department of Surgery, Gastroenterological Center, Yokohama City University, Yokohama, Kanagawa, Japan; 2 Department of Surgery, Yokohama City University, Yokohama, Kanagawa, Japan

**Keywords:** immune-related adverse events, nivolumab, esophageal cancer

## Abstract

**INTRODUCTION:**

Although immune-related adverse events (irAEs) associated with immune checkpoint inhibitors (ICIs) are widely recognized, ICI-related aortitis is very rare and challenging to diagnose.

**CASE PRESENTATION:**

A 70-year-old man with esophageal squamous carcinoma recurring after esophagectomy received paclitaxel therapy after nivolumab therapy. Nivolumab therapy was administered for 26 months; however, no adverse events occurred during treatment. One month after the initiation of paclitaxel therapy, the patient developed a fever lasting for 2 weeks. Thoracoabdominal contrast-enhanced computed tomography (CT) revealed aortic wall thickening and increased fat density in the descending thoracic aorta; however, no other cause of inflammation was detected. Further examination ruled out infectious or autoimmune disease, and the patient was eventually diagnosed with ICI-related aortitis. Methylprednisolone was administered intravenously at a dose of 80 mg, and the fever subsided on the second day after administration. As the inflammation improved, methylprednisolone was switched to oral prednisolone, and the dose was gradually tapered without symptom relapse. Two months after diagnosis, a follow-up CT scan confirmed the resolution of both aortic wall thickening and the increased fat density.

**CONCLUSIONS:**

ICI-related aortitis should be considered as a differential diagnosis for unexplained fever after ICI administration.

## Abbreviations

ASCO
: American Society of Clinical Oncology
CIA
: clinically isolated aortitis
CR
: complete response
CT
: computed tomography
GCA
: giant cell arteritis
irAE
: immune-related adverse event
ICI
: immune checkpoint inhibitors
NCCN
: National Comprehensive Cancer Network

## INTRODUCTION

The development of immune checkpoint inhibitors (ICIs) targeting programmed cell death-1 and cytotoxic T-lymphocyte antigen 4 (CTLA4) has significantly improved the treatment of various cancers. Therefore, the diagnosis and management of immune-related adverse events (irAEs) have also advanced.^1,2)^ The incidence of dermatological, gastroenterological, hormonal, and pulmonary irAEs is relatively common, and the treatment strategy is well established. However, the diagnosis and treatment strategies for rare irAEs, such as ICI-related aortitis remain underdeveloped.

Here, we report a case of ICI-related aortitis due to nivolumab treatment for esophageal squamous cell carcinoma, which was challenging to diagnose and treat.

## CASE PRESENTATION

The patient was a 70-year-old man diagnosed with stage III esophageal squamous cell carcinoma (UtCe cT3N2M0), according to the Japanese Esophageal Society classification.^3)^ He underwent thoracoscopic esophagectomy with D3 lymph node dissection. The pathological diagnosis was UtCe, 30 × 30 mm, type2, SCC, pT3N3M0, pStage IV A; therefore, he received fluorouracil and cisplatin as adjuvant chemotherapy. Five months after completing adjuvant chemotherapy, computed tomography (CT) revealed recurrence in the mediastinal lymph node area (#104R, #106pre). The patient subsequently underwent radiotherapy at a total dose of 59.4 Gy and received two courses of fluorouracil and cisplatin chemotherapy, and achieved a complete response (CR). However, 6 months after chemoradiotherapy, CT showed regrowth of the mediastinal lymph nodes, leading to the initiation of biweekly intravenous nivolumab therapy at a dose of 240 mg per dose as second-line treatment. After 4 cycles of nivolumab therapy, definite tumor shrinkage was observed, and a total of 41 nivolumab cycles were administered. Owing to tumor regrowth, the treatment was switched to third-line intravenous paclitaxel therapy at a dose of 100 mg/m^2^ on days 1, 8, and 15 every 28 days.

**Fig. 1:**
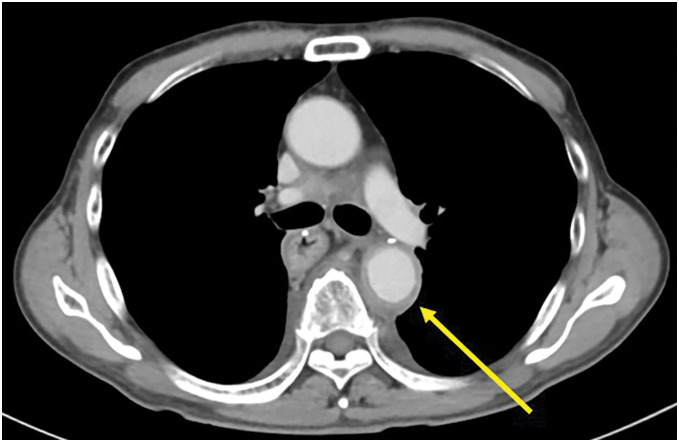
The image of computed tomography taken before the diagnosis of immune checkpoint inhibitor-related aortitis. The image shows wall thickening of the descending thoracic aorta (left arrow).

**Fig. 2:**
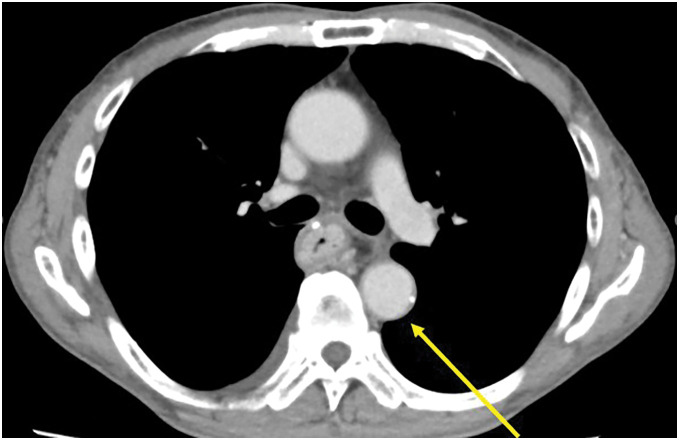
The image of computed tomography taken before nivolumab therapy. The image does not show wall thickening (left arrow).

One month after initiating paclitaxel therapy, the patient developed a persistent fever lasting 2 weeks and visited a local hospital. The patient had no chest or back pain and skin findings characteristic of vasculitis, such as purpura or erythema, were not observed. Although oral antibiotics were administered, the fever persisted, and the patient was referred to our hospital. As the fever did not subside despite continued antibiotic therapy, he was admitted to our hospital for further investigation and treatment. Thoracoabdominal contrast-enhanced CT revealed wall thickening limited to the descending thoracic aorta (**Fig. 1**) compared with that before nivolumab therapy (**Fig. 2**), and blood culture result was negative. Therefore, the patient was diagnosed with nivolumab-induced ICI-related aortitis.

**Fig. 3:**
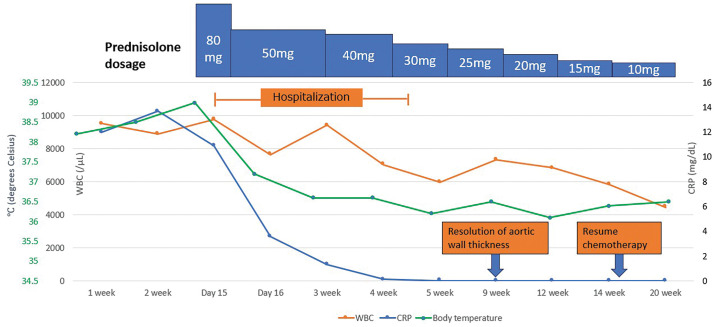
Changes in disease progression since the onset of the symptoms. The vertical axis indicates C-reactive protein (CRP) level, and the horizontal axis represents the number of weeks since symptom onset. After methylprednisolone administration, CRP level decreased immediately.

**Fig. 4:**
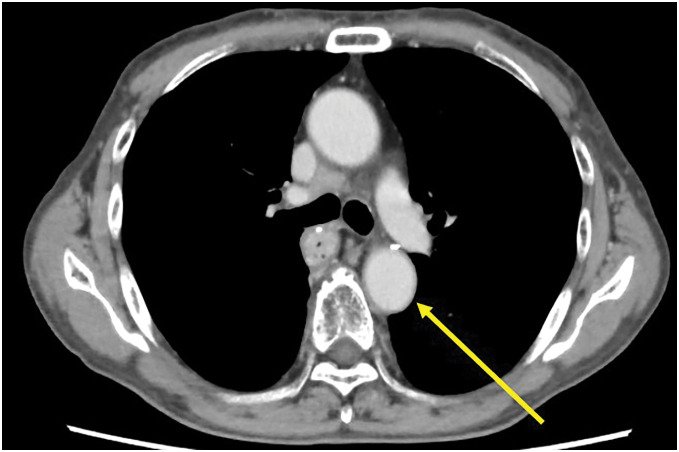
The image of computed tomography taken after the treatment of immune checkpoint inhibitor-related aortitis. The image shows resolution of aortic wall thickening (left arrow).

As shown in **Fig. 3**, methylprednisolone (80 mg) was administered intravenously. Because the fever subsided on the second day, methylprednisolone was switched to oral prednislone, and the dose was gradually decreased to 30 mg. As his general condition and inflammatory response improved, he was discharged on day 15 of hospitalization. Two months after diagnosis, a follow-up enhanced CT showed resolution of aortic wall thickening, as shown in **Fig. 4**, and paclitaxel therapy was restarted while decreasing the prednisolone dose.

## DISCUSSION

Although irAEs associated with ICIs are widely recognized and reported to occur in approximately 13%–14%,^4,5)^ ICI-related aortitis is a rare irAE that remains challenging to diagnose. A single-center retrospective study of 1293 patients receiving ICIs reported only three cases (0.3%) of ICI-related vasculitis.^6)^

To the best of our knowledge, only 4 cases of aortitis associated with irAEs, including our case, have been reported.^7–9)^

**Table 1** shows all 4 case reports of ICI-related aortitis, including cancer type, immunotherapy, treatment used for aortitis, and final clinical outcomes. The types of cancer included squamous cell carcinoma of the lung, melanoma, and esophageal squamous cell carcinoma. The ICIs administered were nivolumab, ipilimumab, and pembrolizumab. The time from immunotherapy initiation to the onset of aortitis ranged from 2 to 27 months.^7,8)^ Every case was treated by discontinuing the ICI and administering either methylprednisolone or prednisolone. One patient was treated with tocilizumab. All cases showed improvement in aortitis. Among the 4 patients, our patient exhibited the latest onset of aortitis, with a 27-month period from nivolumab therapy initiation. While most irAEs occur within 3 months after the initiation of immunotherapy, onset may occur at any time during or after treatment.^2)^ Based on our experience, follow-up for 2 or more years may be recommended.

**Table 1:** Case reports of aortitis as immune checkpoint inhibitors (ICIs)

	Type of cancer	Age	Male/Female	ICI	Immune checkpoint molecule	Time of onset after therapy	Treatment of immune related adverse events	Author
1	Squamous cell carcinoma of the lung	57	M	Nivolumab	PD-1	9 months	Nivolumab discontinued. Methylprednisolone 60 mg IV once. Then the dose is reduced to oral over 4-week period	Roy et al.^7)^
2	Melanoma	51	F	Ipilimumab	CTLA-4	2 months	Prednisone 1 mg/kg/day for 3 weeks	Ban et al.^8)^
3	Melanoma	67	F	Pembrolizumab	PD-1	15 months	High-dose prednisone and tocilizumab	Bloomer et al.^9)^
4	Esophageal cancer	70	M	Nivolumab	PD-1	27 months	Methylprednisolone 80 mg IV, then tapered to oral over 2 weeks	Our case

CTLA4, cytotoxic T-lymphocyte antigen 4

Aortitis is a group of disorders that cause inflammation of the aortic wall. Systemic diseases are associated with non-infectious aortitis that include giant cell arteritis (GCA), Takayasu arteritis, rheumatoid arthritis, systemic lupus erythematosus, Cogan’s syndrome, Behçet’s disease, ankylosing spondylitis, and IgG4-related disease. Infectious aortitis can be caused by bacterial or fungal infections. There are currently no established diagnostic criteria for aortitis as an irAE. In our case, all examinations, including complete blood count, erythrocyte sedimentation rate, C-reactive protein, rheumatoid factor, antinuclear antibodies, antineutrophil cytoplasmic antibodies such as p-ANCA and c-ANCA, complement levels, and blood culture, were negative. One differential diagnosis in such cases is Takayasu arteritis, which can sometimes be detected incidentally in the early phase. However, Takayasu arteritis usually affects younger women and often shows characteristic imaging findings such as concentric wall thickening of multiple large vessels. Based on the clinical presentation and negative workup for other causes, the patient was diagnosed with nivolumab-induced ICI-related aortitis.

Although the treatment of vasculitis is not clearly discussed in the American Society of Clinical Oncology or National Comprehensive Cancer Network guidelines, steroids were administered and tapered according to treatment protocols for peripheral vasculitis caused by GCA or other irAEs.^10)^ In prednisolone-resistant cases, the administration of immunosuppressants might be an alternative treatment.^11–13)^

Managing irAEs such as aortitis can be particularly challenging in patients undergoing cancer treatment. Although steroid therapy is commonly used for irAEs, there is limited clinical evidence about how well it works, especially for vascular inflammation.

It remains unclear how immunosuppressive agents like methotrexate or tocilizumab should be used in cases that are resistant to steroids or show recurrence, which is a clinical concern.

## CONCLUSIONS

ICI-related aortitis should be considered a differential diagnosis for unexplained fever after ICI administration.

## DECLARATIONS

### Funding

This research did not receive any specific grant funding.

### Authors' contributions

NA, SS, KS, and TS wrote the first draft of this manuscript.

All authors provided feedback on previous versions of the manuscript and have read and approved the final manuscript.

### Availability of data and materials

The data and materials used in this case report are not publicly available due to patient privacy concerns.

### Ethics approval and consent to participate

This work does not require ethical considerations or approval. Informed consent to participate in this study was obtained from the patient.

### Consent for publication

Verbal consent was obtained from the patient for publication of the details of this case and any accompanying images.

### Competing interests

The authors declare that they have no competing interests.

The disclosed honoraria are not directly associated with the content or findings presented in this case report.
